# Genome assembly of antimicrobial-resistant *Escherichia coli* HMVC1 isolated from healthy Mogosane village cattle, South Africa

**DOI:** 10.1128/mra.01273-23

**Published:** 2024-02-22

**Authors:** Lerato Lisbeth Njaki Makhetha, Baitsholetsi Gloria Mokolopi, James Wabwire Oguttu, Christian Anayochukwu Mbajiorgu, Goitsemang Makete, Tshifhiwa Paris Mamphogoro

**Affiliations:** 1 Department of Agriculture and Animal Health, University of South Africa, Science Campus, Florida, South Africa; 2 Gastro-Intestinal Microbiology and Biotechnology Unit, Agricultural Research Council-Animal Production, Irene, Pretoria, South Africa; University of Maryland School of Medicine, Baltimore, Maryland, USA

**Keywords:** *Escherichia coli*, cattle, antimicrobial resistance genes, genome assemblies

## Abstract

Here, we present the genome assembly of *E. coli* strain HMVC1 isolated from rectal fecal samples of healthy cattle in South Africa. The genome size of HMVC1 consisted of 5,043,843 bp, with G + C content of 50.5%. The strain harbors *mar*A, *mdt*M, *acr*F, *acr*D, and other antimicrobial resistance genes.

## ANNOUNCEMENT

*Escherichia coli* in farm animals has extensively been studied for decades in developed countries, and its data on whole genomic sequencing (WGS) are stored in the GenBank. Developing countries such as South Africa still have more work regarding depositing WGS of *E. coli* isolates from cattle in the GenBank for future studies (1). Besides the reputation of some strains of *E. coli* as animal pathogens, *E. coli* has been shown to account for the majority of resistance in *Enterobacteriaceae*; this bacterium has been postulated to serve as a reservoir of antimicrobial resistance genes within the digestive tract (2).

*E. coli* HMVC1 was isolated from rectal fecal samples of healthy cattle in Mogosane Village in the North West province, South Africa (25°45′30.6″S 25°33′43.9″E). A loopful of feces sample was inoculated into Buffered Peptone Water medium; the mixture was serially diluted up to 10^−2^ and inoculated onto McConkey agar for 24 h at 37°C. The pure culture was obtained by repeated streaking onto sterile Blood Agar (BA) (3). Genomic DNA was isolated from overnight culture on BA using HighPure PCR template preparation kit (Roche Diagnostics, Mannheim, Germany), in accordance with the manufacturer’s instructions. A NanoDrop (ThermoFisher Scientific, Carlsbad, CA, USA) was used to determine the concentration of extracted DNA, while the quality of the DNA was assessed using a 2% agarose gel. The DNA libraries were generated employing the Illumina TruSeq DNA Nano Preparation Kit (Illumina, San Diego, CA, USA); these libraries were sequenced by 150 bp paired-end sequencing on Illumina Hiseq X machine, producing a total of 4,845,267 paired-end 2 × 150 bp reads. The raw reads were trimmed using Trimmomatic v0.36 (4) and quality controlled using FastQC v0.11.5 (5). Trimmed reads were then *de novo* assembled using SPAdes v3.13.0 (6). Assembly quality was evaluated using QUAST v5.0.2 (7). While the genome completeness and contamination were assessed using CheckM v1.0.18 (8).

Identification of HMVC1 was conducted using Kaiju v1.7.3 (9), and the results were visualized using Krona v2.7.1 (10). Annotation was performed using the NCBI Prokaryotic Genome Annotation Pipeline (PGAP) v6.3 (11). Default parameters were used for all software unless otherwise specified. The HMVC1 genome size consisted of 5,043,843 bp and is predicted to contain 4,960 total genes, of which 4,663 are predicted to encode proteins. Genome completeness was estimated at 100%, consisting of 110 contigs and the G + C content of 50.5%. The *N*_50_ value was 141,930 bp with a genome coverage of 354×. Antimicrobial-resistant genes were screened using resistant gene identifier (RG1) v5.1.1, which employs the comprehensive antibiotic resistance database v3.1.0 (12), allowing a broad homology-based search with defined criteria ranging from “Perfect” to “Strict” matches. A total of 38 strict hits along with 15 perfect were detected. *E. coli* HMVC1 harbor genes providing resistance to fluoroquinolones, cephamycins, tetracyclines, rifamycins, and penams along with that the resistance mechanisms mainly antibiotic efflux, reduced permeability, and antibiotic target alteration (Table 1) (13, 14). This study marks the ability of the isolated bacterial strains to be used in the production of novel antimicrobial compounds and in the surveillance of antimicrobial drug resistance.

**TABLE 1:** (A) Characteristics of the sequenced *Escherichia coli* HMVC1 and (B) antibiotic-resistant genes encoded by the strain

(A) Characteristics of the sequenced ***E. coli*** HMVC1					
Strain name	Date of Isolation	Isolation source	Number of rRNAs	Number of tRNAs	Number of RNAs
HMVC1	14/06/2017	MVNWPSA^*a*^ (healthy Bos Taurus fecal sample)	7	76	91

(B) Antibiotic resistant genes in *E. coli* HMVC1					
Criteria used by RGI^*b*^	AROTerm^*c*^	Family of AMR genes^*d*^	Drug class	Mechanisms of resistance	Identity with the matching region (%)
Perfect	*mdt*N	Major facilitator superfamily (MFS) antibiotic efflux pump	Nucleoside antibiotic, disinfecting agents, and antiseptics	Antibiotic efflux	100.0
Perfect	H-NS	MFS antibiotic efflux pump, resistance-nodulation-cell division (RND) antibiotic efflux pump	Macrolide antibiotic, fluoroquinolone antibiotic, cephalosporin, cephamycin, penam, tetracycline antibiotic	Antibiotic efflux	100.0
Perfect	*tol*C	ATP-binding cassette (ABC) antibiotic efflux pump, MFS antibiotic efflux pump, RND antibiotic efflux pump	Macrolide antibiotic, fluoroquinolone antibiotic, aminoglycoside antibiotic, carbapenem, cephalosporin, glycylcycline, cephamycin, penam, tetracycline antibiotic, peptide antibiotic, aminocoumarin antibiotic, rifamycin antibiotic, phenicol antibiotic, penem, disinfecting agents, and antiseptics	Antibiotic efflux	100.0
Perfect	*mdt*E	RND antibiotic efflux pump	Macrolide antibiotic, fluoroquinolone antibiotic, penam	Antibiotic efflux	100.0
Perfect	*mdt*H	MFS antibiotic efflux pump	Fluoroquinolone antibiotic	Antibiotic efflux	100.0
Perfect	*mdt*G	MFS antibiotic efflux pump	Phosphonic acid antibiotic	Antibiotic efflux	100.0
Perfect	*mar*A	RND antibiotic efflux pump, General Bacterial Porin with reduced permeability to beta-lactams	Fluoroquinolone antibiotic, monobactam, carbapenem, cephalosporin, glycylcycline, cephamycin, penam, tetracycline antibiotic, rifamycin antibiotic, phenicol antibiotic, penem, disinfecting agents, and antiseptics	Antibiotic efflux, reduced permeability to antibiotic	100.0
Strict	*mdt*O	MFS antibiotic efflux pump	Nucleoside antibiotic, disinfecting agents, and antiseptics	Antibiotic efflux	98.54
Strict	*mdt*M	MFS antibiotic efflux pump	Fluoroquinolone antibiotic, lincosamide antibiotic, nucleoside antibiotic, phenicol antibiotic, disinfecting agents, and antiseptics	Antibiotic efflux	98.29
Strict	*acr*D	RND antibiotic efflux pump	Aminoglycoside antibiotic	Antibiotic efflux	99.9
Strict	*Acr*F	RND antibiotic efflux pump	Fluoroquinolone antibiotic, cephalosporin, cephamycin, penam	Antibiotic efflux	99.52
Strict	*E. coli sox*R with mutation conferring antibiotic resistance	ABC antibiotic efflux pump, MFS antibiotic efflux pump, RND antibiotic efflux pump	Fluoroquinolone antibiotic, cephalosporin, glycylcycline, penam, tetracycline antibiotic, rifamycin antibiotic, phenicol antibiotic, disinfecting agents, and antiseptics	Antibiotic target alteration, antibiotic efflux	100.0
	*E. coli sox*S with mutation conferring antibiotic resistance	ABC antibiotic efflux pump, MFS antibiotic efflux pump, RND antibiotic efflux pump, General Bacterial Porin with reduced permeability to beta-lactams	Fluoroquinolone antibiotic, monobactam, carbapenem, cephalosporin, glycylcycline, cephamycin, penam, tetracycline antibiotic, rifamycin antibiotic, phenicol antibiotic, penem, disinfecting agents, and antiseptics	Antibiotic target alteration, antibiotic efflux, reduced permeability to antibiotic	100.0
Strict	*emr*K	MFS antibiotic efflux pump	Tetracycline antibiotic	Antibiotic efflux	99.43

^*a*^ MVNWPSA, Mogosane Village in the North West province in South Africa.

^*b*^ RGI, Resistant Gene Identifier.

^*c*^ ARO, Antibiotic Resistance Ontology.

^*d*^ AMR, Antimicrobial Resistant.

## Data Availability

This whole-genome shotgun project has been deposited at DDBJ/ENA/GenBank under the accession number JAWJYW000000000. (The version described in this paper is the first version. The SRA accession number is SRR26374259, the BioProject accession number is PRJNA1028407.)
